# 625. Closing Knowledge Gaps in Flammable Liquid Safety: Results of a Community Survey and Prevention Campaign

**DOI:** 10.1093/jbcr/irag033.451

**Published:** 2026-04-06

**Authors:** Ginger E Leonard, James H Holmes, IV, Cynthia J Mastropieri, Christopher K Craig

**Affiliations:** Wake Forest Baptist Medical Center Burn Center, Mocksville, NC; Atrium Health Wake Forest Baptist Burn Center, Winston-Salem, NC; Atrium Health Wake Forest Baptist, Winston-Salem, NC; Atrium Health Wake Forest Baptist Hospital / High Point University, High Point, NC

## Abstract

**Introduction:**

Flammable and combustible liquids are a major cause of residential fires, contributing to more than 51 000 incidents annually in the country, with 169 deaths, 1029 injuries, and $644 million in property damage. Gasoline, in particular, poses significant risks when stored or used unsafely. In addition, careless open burning of debris, yard waste, and bonfires, often accelerated by flammable liquids, remains a common mechanism of severe burn injury. State laws restrict open burning practices, yet compliance and awareness are inconsistent. This project aimed to evaluate public knowledge, attitudes, and behaviors regarding flammable liquid use, with the goal of informing a multifaceted community education and prevention campaign aligned with the ABA National Burn Awareness Week.

**Methods:**

A 12 item survey was developed in REDCap to assess public knowledge and behaviors regarding flammable liquids. The survey was distributed via QR code on social media, hospital bulletins, a regional trauma organization website, and at community outreach events featuring a flammable liquid education booth. Educational resources provided included statistics on fire and burn risks, safe handling practices, state forest service contact numbers, public service announcements, burn first aid tips, and incentives such as flame retardant blankets and fire extinguishers. The initiative was conducted in partnership with the city fire department.

**Results:**

A total of 125 individuals completed the survey, 73% of whom were female. Nearly one in five respondents (19%) reported sustaining a burn injury related to flammable liquid use. Knowledge of key safety practices varied: 89% knew to contact the fire department prior to burning brush, 87% correctly identified gasoline, paint thinner, and turpentine as highly flammable, 71% reported storing flammable liquids in labeled containers, and 70% were aware that oil soaked rags can spontaneously combust. In contrast, only 56 to 60% reported awareness of burn permit requirements and the associated financial penalties for noncompliance.

**Conclusions:**

This survey identified important gaps in public knowledge regarding flammable liquid safety, particularly in areas related to permitting and burn restrictions. A multifaceted prevention campaign that integrates structured education, community partnerships, and targeted messaging was associated with strong public engagement and provides a framework for ongoing injury prevention.

**Applicability of Research to Practice:**

Burn centers, trauma programs, and community fire safety organizations can replicate this model by combining survey based needs assessment with outreach and education tailored to identified knowledge gaps. Sustained engagement through public campaigns such as #STOPTHINKFIRST, #BewareFlammableLiquids, and #DontBeCareless may help reduce injuries and fatalities from unsafe use of flammable liquids.

**Funding for the study:**

N/A.

